# Categorical perception and language evolution: a comparative and neurological perspective

**DOI:** 10.3389/fpsyg.2023.1110730

**Published:** 2023-04-27

**Authors:** Elizabeth Qing Zhang, Edward Ruoyang Shi, Lluís Barceló-Coblijn

**Affiliations:** ^1^School of Linguistic Sciences and Arts, Jiangsu Normal University, Xuzhou, Jiangsu, China; ^2^Center for Language Evolution Studies, University Centre of Excellence IMSErt, Nicolaus Copernicus University, Toruń, Poland; ^3^Department of Translation and Language Sciences, Universitat Pompeu Fabra, Barcelona, Spain; ^4^Laboratori d'Investigació en Complexitat i de Lingüística Experimental, Palma de Mallorca, Spain; ^5^Laboratori d'Investigació en Complexitat i de Lingüística Experimental, Universitat de les Illes Balears, Illes Balears, Palma, Spain

**Keywords:** categorical perception, categorical learning, discreteness, comparative perspective, neurological perspective, language evolution

## 1. Introduction

Categorical Perception (CP) is a ubiquitous phenomenon in nature (Eimas et al., 1971; Goldstone and Hendrickson, 2010). Discreteness is a prominent feature of human language (Hockett, 1960). In this paper, we propose that CP could have played a foundational role for discreteness of language in evolution. We firstly approach discreteness from a domain general perspective and highlight how it is salient in language. Then by reviewing CP of sounds in non-human animals, we argue that CP has its phylogenetic roots in terms of evolution. Following this, we explain how CP could have been the basis for discreteness with neurological evidence focusing on the auditory cortex, (pre)motor cortex and the basal ganglia. At last, we suggest that clinical linguistics provides revealing insights on the role of CP in language. The current work discusses the role of perception in language evolution, which provides a new avenue to explore the evolution of human language from the sensory-motor system.

## 2. Discreteness

Discreteness is an essential concept in quantum physics, chemistry, mathematics, and human cognition (Abler, 1989). Discrete and compositional language differs from continuous and holistic non-human animal communication signals, which is one of the design features of human language (Hockett, 1960), in the sense that phonemes of a language are contrastive. For example, in English, “pin” and “bin” differ only on the voice onset time of the initial plosives of the words that changing the onset of a syllable alters the meaning of the words. In other words, phonemes of syllables can be reused for new syllable composition. This is in contrast to non-human animal signals, which are fixed combinations of sounds. What Hockett has emphasized is discreteness observed in the phonological structure of language, namely syllables are made up of separate vowels and consonants. This is directly linked to *duality of patterning*, another of Hockett's language features, according to which phonemes are combined in order to codify meaning, being the morphemes the result of such a codifying process. It has been observed that discreteness is a prerequisite for coding, and that, during evolution, duality of patterning would have been favored as soon as (proto)language was a discrete system (Fortuny, 2010). Discreteness is also observed at syntactic levels. Lexical elements are discretely dispersed and organized into grammatical phrases or sentences based on the rules.

## 3. Categorical perception and categorical learning

Categorical perception (CP) is a psychophysical process in which continuous inputs are perceived discretely across modalities (Harnad, 1987). The interactions between low-level perception and high-level cognition are revealed by this implicit segmentation of continuous physical inputs (Goldstone and Hendrickson, 2010). The phenomenon of CP was initially identified in human speech sounds (Liberman, 1957). Listeners were inclined to perceive the b-d-g continuum into three distinct groups. Zhang et al. (2021) argued that CP is a combination of nature and nurture. Research on infants has revealed that they appear to be endowed with the ability to discriminate different sounds in all languages (which is the nature part), but as they have more contact with one (or more for multilinguals) ambient language(s), they tend to group sounds that are not contrastive in their native language(s) (Werker and Tees, 1984; Kuhl et al., 2006). By postnatal categorical learning (Livingston et al., 1998), the between-and within-category boundaries will be acquired and change corresponding to the environment (Pérez-Gay et al., 2017; Pérez-Gay Juárez et al., 2019).

## 4. Categorical perception and discreteness: comparative evidence

It is worth emphasizing that CP is a phenomenon across domains and modalities (Burns and Ward, 1978; Goldstone and Hendrickson, 2010). If our hypothesis that discreteness arises from CP is correct, the discovery of CP in non-human animals shows that discreteness of language may have been derived from a preserved trait, namely CP. Comparative studies in non-human animals reveal that CP is phylogenetically anchored in early invertebrates, which could be the evolutionary origin of the nature part of CP (Zhang et al., 2021). In this section, we present examples of CP in the sound modality in non-human animals.

Crickets have been observed to be able to distinguish between communication calls and predator ultrasounds at a sharp border when it comes to naturally produced noises (Wyttenbach et al., 1996). Female túngara frogs respond to mating sounds in a categorical manner (Baugh et al., 2008). Non-human primates were shown to be able to discriminate both consonants and vowels (e.g., Sinnott and Mosteller, 2001). In birds, it was discovered as early in the 1980's that budgerigars not only had a low discrimination frequency corresponding to their contact calls, but also had a similar range of voice onset time to humans in the perceptual change of bilabial, alveolar, and velar continua (Dooling et al., 1987). The budgerigars used the same cues as humans to distinguish between vowel groups (Dooling and Brown, 1990). These data suggest that CP probably has its phylogenetic origin from early invertebrates.

Returning to our hypothesis that CP prepared the basis for discreteness, it has been shown that the discreteness of sounds and the discreteness of words are inextricably intertwined. When it comes to CP of speech sounds, or how to discriminate and categorize speech sounds in a given language, it appears that sequential statistics manifested in words (Transitional Probability), rather than acoustic features of the sounds, drive discrimination and categorization of the speech sounds (Saffran et al., 1996). Furthermore, in written language, CP has been shown to have effects in Chinese character perception (Yang and Wang, 2018). Data in infants suggest that perceptual statistical learning also plays a key role in word segmentation from speech streams (Romberg and Saffran, 2010). In this sense, the key of discreteness offered at the phoneme level appears to be influenced by how sound sequences in words are organized (Bidelman and Lee, 2015). Moreover, it has been shown that category learning and word learning are closely related. If labels are given to new categories, they will be easier to learn (Zettersten and Lupyan, 2020). These indicate that discreteness may have been founded on categorical perception.

## 5. Categorical perception and discreteness: neurocognitive considerations

Neurobiological studies on CP and discreteness of language also support our proposal. In birds, HVCx [HVC (a letter-based term) projects to AreaX (striatal area x)] cells in swamp sparrows have been demonstrated to respond robustly to auditory categorical changes in note duration (Prather et al., 2009). HVC is a premotor nucleus analogous to Broca's area in humans and serves sensory-motor functions (Prather et al., 2017). HVCx could be analogous to the premotor-striatal connection, which has been linked to beat perception in humans (Grahn and Rowe, 2009). In European Starlings, the auditory nuclei field L projecting to CLM (caudolateral mesopallium) and NCM (the caudal part of the medial nidopallium), both projecting to CMM (the caudal part of the medial mesopallium), present an analogous hierarchy to humans in which physical information is processed at the lower level while abstract concepts are encoded at the higher level (Jeanne et al., 2011). NCM and CMM are similar to the human auditory cortex for auditory memory (Bolhuis and Gahr, 2006). NCM and CMM in zebra finches are more responsive to rhythmic than arrhythmic songs (Lampen et al., 2017), indicating that both are involved in auditory detection and discrimination.

The auditory cortex is also involved in CP of speech sounds in primates. Spiking activity from the superior temporal gyrus (STG) in rhesus monkeys were recorded (Tsunada et al., 2011). In humans, CP of sounds was mediated by the STG and superior temporal sulcus (STS) in the tasks of phonemic and non-phonemic discrimination (Harinen and Rinne, 2013). In addtion, Premotor cortex (PMC) is activated in phoneme categorization tasks (Chevillet et al., 2013) while primary motor cortex is found to be involved in the study of CP of speech sound (Möttönen and Watkins, 2009). The dorsal language stream connects the STG with the premotor cortex, responsible for sensorimotor transformation in speech output (Hickok and Poeppel, 2004). Both STG and premotor cortex were reported to be where CP takes place. If CP is the basis for discreteness, such dorsal connection could have played a role in discreteness. The dorsal pathway has been shown to be necessary for vocal imitation, which seems one of the key factors for word emergence (Edmiston et al., 2018). Structurally, such connection between STG and PMC (dorsal pathway I) serves as the basis for dorsal pathway II for complex syntactic processing during development (Brauer et al., 2011). In this sense, discreteness seems to rely on CP at the phonological level (phonemes) which in turn lays the foundation for CP at the syntactic level (words).

Beyond cortex, recent evidence has shown that the basal ganglia also relate to perceptual categorization (Seger, 2008). Basal ganglia-mediating category learning and speech perception and learning have provided great potential in bridging efforts to understand speech perception and learning with general cognitive neuroscience approaches and neurobiological models of Learning (Lim et al., 2014). The basal ganglia (i.e., the striatum) take part in category training of non-native speech categories (Tricomi et al., 2006). These results suggest that the basal ganglia learning system are involved in promoting adult speech category learning. This is also in parallel with the finding in swamp sparrow mentioned above that Area X is also involved in CP of note duration.

## 6. Categorical learning: from clinical linguistic perspective

Ashby and Ell (2001) point out that category-learning tasks can be either rule-based (for example, by means of the Wisconsin Card Sorting Test), or based on information-integration, or prototype-distortion tasks. Individuals suffering from a disease in the basal ganglia (e.g., Huntington's (HD) or Parkinson's disease have impaired the basal ganglia and seem to have more problems in rule-based tasks and information-integration tasks (Knowlton et al., 1996). Rao et al. (1997) in their work on rule-based tasks provided fMRI data showing the participation of the cerebral cortex, basal ganglia, thalamus, and cerebellum in conceptual reasoning tasks. Cope et al. (2014) put to the test three groups of participants, the two first affected by Multiple System Atrophy, and the third affected by HD in tasks of perceptual timing and stimuli were pure tones of different duration. Results showed that HD participants had more severe impairments, leading to the conclusion that basal ganglia are a “mandatory component for absolute, duration-based as well as relative, beat-based timing.”

While classic works on Williams Syndromes and cognition initially suggested a relatively spared–perhaps even modular– language capability (Bellugi et al., 1994; Pinker, 1995), other scholars have suggested that a perspective of ontogenetic development does not seem to support such a modular view (Stojanovik et al., 2004; D'Souza and Karmiloff-Smith, 2011). The integration of semantic information into sentence comprehension (Tyler et al., 1997), and the semantic organization of concepts seems not typical (Jarrold et al., 2000). Moreover, WS speakers seem to produce more errors in semantic categorization tasks (Purser et al., 2011). If we consider the mechanism underlying the connection between language and other domains, the impairment of CP and categorical learning in this group of patients could give rise to discreteness related symptoms. Children with WS have difficulty in segmenting the speech stream (Brock, 2007), and have an atypical sensitivity toward subtle acoustic variations during speech and non-speech auditory analysis (Majerus et al., 2011). At the neural level, WS patients have basal ganglia atrophy (Faria et al., 2012), and disproportional reductions occur in the putamen and nucleus accumbens which can predict WS status (Fan et al., 2017). While fMRI analyses point out abnormalities characterized by a reduction of gray matter volume in cortical areas (Osório et al., 2014) and the basal ganglia –including the caudate nucleus, basal ganglia and thalamus—(Campbell et al., 2009; Jackowski et al., 2009; Meda et al., 2012; Hanson et al., 2018).

In the autism spectrum disorder (ASD), problems in the connections between basal ganglia, cerebral cortex and the cerebellum may give rise to problems in various motor and cognitive processes (Subramanian et al., 2017). It has been assumed that language impairment in autistic children could serve as a good indicator of their inability to categorically perceive speaking sounds (Rong et al., 2022). Furthermore, language proficiency in Mandarin-speaking ASD was positively associated with a greater degree of CP of lexical tones (Chen and Peng, 2021).

Evidence reviewed in this section is consistent with the discussion of the involvement of the basal ganglia in CP and categorical learning. We suggest that clinical linguistics could shed light on the relation between language and other cognitive domains.

## 7. Conclusion

In this paper, we come up with the hypothesis that categorical perception (CP) could have laid the foundation for discreteness, one of the design features of language. By reviewing comparative studies on CP in non-human animals and humans, we found that CP has a phylogenetic root dating back to invertebrates which is closely related to reproduction and survival, and seems to be a combination of innateness and experience. The result of category learning creates or changes CP in experience. In addition, by reviewing neurobiological studies, we show that tasks of CP activate cortical and subcortical areas including auditory and (pre)motor cortex as well as the basal ganglia, the connection between which could be insightful for locating domain general discreteness. We also point out that there are CP and category learning related problems in several clinical conditions. The current work provides additional evidence for the important role of the sensory-motor system in language evolution.

## Author contributions

EZ conceptualized and wrote and revised the paper. ES wrote the categorical learning part and revised the paper. LB-C wrote the clinical linguistic part and revised the paper. All authors contributed to the article and approved the submitted version.
